# Burnout and motivation to study medicine among students during the COVID-19 pandemic

**DOI:** 10.3389/fmed.2023.1214320

**Published:** 2023-08-28

**Authors:** Clio Metakides, Lena Pielemeier, Theodore Lytras, Dimitrios G. Mytilinaios, Sophia C. Themistocleous, Chryso Pieridi, Constantinos Tsioutis, Elizabeth O. Johnson, Dimitrios Ntourakis, Ilias P. Nikas

**Affiliations:** ^1^School of Medicine, European University Cyprus, Nicosia, Cyprus; ^2^Kenhub GmbH, Leipzig, Germany

**Keywords:** burnout, motivation, online education, emergency remote teaching, medical school, COVID-19, pandemic

## Abstract

**Aim:**

To investigate medical students’ burnout and motivation levels in each of the six years of their studies during the COVID-19 pandemic and identify independent predictors of burnout and motivation.

**Methods:**

An anonymous cross-sectional survey was sent to the students of all six years within our school. Burnout was measured with the adapted Oldenburg Burnout Inventory questionnaire (OLBI-S) and motivation with the updated Strength of Motivation for Medical School (SMMS-R) questionnaire. Univariate analysis was performed with the Kruskal–Wallis test and Spearman’s correlation, while multivariable analysis with linear regression models.

**Results:**

A total of 333 medical students (52% of student body) responded. Higher burnout levels correlated with lower motivation to study medicine (rho = −0.30, *p* < 0.001). Burnout levels differed between the six years of medical studies, peaking in years two and four whereas being the lowest in year one (*p* = 0.01). Motivation levels differed significantly as well, peaking in years one and four whereas being the lowest in years five and six (*p* = 0.012). In the multivariable linear regression models, being a female (*b* = 2.22, *p* = 0.016), studying in the fourth year vs. first year (*b* = 2.54, *p* = 0.049), having a perceived beginner/intermediate vs. advanced/expert technology level (*b* = 2.05, *p* = 0.032) and a perceived poor school support system (*b* = 6.35, *p* < 0.001) were independently associated with higher burnout levels. Furthermore, studying in the fifth year vs. first year (*b* = −5.17, *p* = 0.019) and a perceived poor school support system (*b* = −3.09, *p* = 0.01) were independently associated with a reduced motivation to study medicine.

**Conclusion:**

Our study highlighted potential areas for intervention to decrease the rate of burnout and low motivation among medical students. However, further research is needed to unravel the full effect of the pandemic on medical students.

## Introduction

According to the World Health Organization (WHO), burnout is defined as an “occupational phenomenon resulting from chronic workplace stress that has not been successfully managed” (1). Burnout is characterized by three main components: (a) being exhausted or lacking energy, (b) feeling negative and mentally distant towards the occupation, and (c) having a decreased professional efficacy (1). In the academic settings, burnout could lead to depression or suicidal ideation; it is noteworthy that approximately half of the medical students within the United States have reported to be suffering from burnout at some point in their medical education (2).

Motivation of students determines their academic success and endurance in their specific field of education (3). Different types of motivation comprise the extrinsic motivation – resulting from external factors, such as rewards for achievements – and intrinsic motivation, for example altruism, arising from within (4). For medical students in particular, motivation might differ from students in other majors, as their learning environment with an increased number of practical laboratories and the additional presence of clinical training is rather unique and demanding (3). Furthermore, due to the highly competitive admission requirements of many medical schools, students are thought to exhibit increased levels of motivation to apply and get accepted, compared to students in other education fields (3). Few studies have so far investigated the motivation of medical students. Győrffy et al. conducted a study with medical students regarding their career choice motivation and found that cynicism as well as decreased levels of academic efficacy, both components of burnout, were related to paucity of altruistic motivation (4). In addition, Kusurkar et al. completed a study analyzing medical students’ motivation profiles and reported the best learning profile in the ones with high motivation types, whereas students with lower motivation exhibited less desirable learning behaviors (5).

Coronavirus disease 19 (COVID-19) caused a global pandemic and affected all levels and areas of education, transforming medical school teaching worldwide (6, 7). In order to limit the spread of COVID-19 and still allow education to continue without interruption, many medical schools implemented emergency remote teaching (ERT), whereas they heavily modified their mode of preclinical instruction and clinical placements in their healthcare settings (6, 8, 9). Despite a few positive implementations largely within the preclinical education context (8–11), medical students worldwide often perceived remote medical education negatively, especially during their clinical years, while they also reported ERT impacted their mental health negatively. For instance, Nikolis et al. found that the overall wellness of medical students decreased, according to their survey of 1,389 medical students in the US (12). Al-Balas et al. conducted a study in Jordan at the beginning of the pandemic and reported that just one out of four students were satisfied with their medical education (13). Singh et al. noted that more than half of the responders enrolled in the All India Institute of Medical Sciences would favor in-person rather than at-distance classes (14). Notably, Franklin et al. surveyed their senior medical school year and reported that students perceived remote education as less effective compared to traditional physical hospital rotations and didactic face-to-face lectures (15). Chakladar et al. reported an alarming number of medical students struggling with anxiety regarding their future, also depression and second thoughts regarding their future profession during the COVID-19 pandemic, indicating how crucial it is for medical schools to address these problems or provide students with the necessary resources (16). Lastly, a systematic review from Finland investigated the clinical learning experience of culturally and linguistically diverse healthcare students and found out that this particular group of students experienced clinical integration as stressful (17). It is noteworthy that the student body of our medical school represents a culturally and linguistically diverse population with most students originating from foreign countries inside and outside the EU. In addition, our school’s curriculum is in English, whereas the mother language of the vast majority of our students is not.

Considering the challenge of admission in combination with the demanding learning environment of medical school, it is apparent that the added stress of a global pandemic affecting daily life as well as the shift to remote learning could have impacted the students’ burnout levels, motivation, and ultimately their academic performance. Whereas burnout among medical students during this period has largely been studied mainly by using the Maslach Burnout Inventory Student-Survey (MBI-SS) (18–20), the pandemic’s effect on motivation to study medicine remains elusive. This study aimed to assess the students’ burnout and motivation levels in our medical school in each of the six years of their studies, also to identify independent predictors of burnout and motivation to study. Regarding burnout and instead of the MBI-SS, we used another scale less often reported, albeit already validated and applicable to our study population, the Oldenburg Burnout Inventory questionnaire adapted for academic environments (OLBI-S) (21).

## Methods

This cross-sectional study received approval from the Cyprus National Bioethics Committee (2021.01.79). An optional and anonymous survey was delivered electronically to the students of all 6 years within our medical school via the Google Forms platform (Google LLC., Mountain View, CA). The survey stayed open for responses from April 12 until May 21, 2021, while students were notified with weekly reminders through email and the social media group of their study year. During this period and because of the pandemic, some classes were being conducted remotely, while others in a hybrid fashion (online and on-campus in small teams). Our medical school runs a 6 year program, following a spiral and competency-based curriculum, while the classes are being delivered in the English language (8). The survey included a selection of demographic, lifestyle, and course delivery preference questions (on-campus, online or blended); the latter were delivered only to years 2–6, as our first-year medical students had no experience of on-campus medical education during this time. In addition, it included two scales related to burnout and motivation to study medicine, composed of Likert items. To measure the burnout levels of our students, the OLBI-S was used; this validated scale contains 16 questions with answers ranging from 1 (strongly agree) to 4 (strongly disagree) and includes two subscales, “exhaustion” and “disengagement,” with a total score from 16–64 (21). To measure motivation, the updated Strength of Motivation for Medical School (SMMS-R) questionnaire was used; this validated scale contains 15 questions with answers ranging from 1 (strongly disagree) to 5 (strongly agree) and includes three subscales (“willingness to sacrifice,” “readiness to start,” and “persistence”), with a total score from 15–75 (22).

The data of this e-survey were exported into an Excel sheet (Microsoft Corporation, Redmond, WA) and subsequently underwent statistical analysis using the R software (R Foundation for Statistical Computing, Vienna, Austria). Continuous variables were expressed in medians with their interquartile ranges (IQR). Medians were compared with the Kruskal–Wallis test, whereas the correlations between burnout and motivation (including their subscales) with the Spearman’s test. Lastly, two multivariable linear regression models were defined to identify independent predictors of higher burnout levels and motivation to study among medical students during the COVID-19 pandemic. Both models included *a priori* the following covariates: sex, year of study (as categorical variable), mother language, self-reported level of familiarity with technology, financial concerns, personal health issues, extracurricular responsibilities and perceived level of school support. Significance levels were defined at *p* < 0.05.

## Results

A total of 333/641 (52%) students from all 6 years of our medical school program during this period participated in the survey. Firstly, our study revealed a significant association of student higher burnout levels with lack of physical exercise, smoking, poor food quality (e.g., fast food or microwave meals), weight gain, poor sleep quality, higher social media use, and shorter preparation time for classes. Daily alcohol consumption showed only a tendency of higher burnout when compared with occasional use. Furthermore, the following variables related to the changes brought by the COVID pandemic were significantly associated with higher burnout levels in our students: perceived lower level of familiarity with technology, deteriorating financial situation, serious health issues or negative events in personal life, extracurricular responsibilities, and a perceived poor support from the medical school faculty/advisors (Supplementary Table S1).

Motivation was not generally affected by the same factors as burnout with the exception of the student’s mother language, extracurricular responsibilities, and perceived quality of support received from faculty. However, important interactions between students’ preference concerning the mode of course delivery (online, blended or on-campus) and their motivation were found. Students who preferred an online only curriculum consistently had lower motivation in comparison to students who preferred on-campus activities or did not have a preference (Table 1).

**Table 1:** Univariate analysis of the effect of student and curriculum related factors on medical student motivation during the COVID-19 pandemic.

Variable	Motivation SMMS-R (Full Scale)			
	Categories	Median	IQR	*p*-value
Mother language	Greek	59	53–64	0.042
	English	62	54.5–71	
	German	55	47.25–63	
	Other	59.5	53.75–68	
Extracurricular responsibilities	Yes	58	50.5–64.5	0.04
	No	60	55–66	
Perceived support from faculty/advisors	Very good	64	59.5–70.5	<0.001
	Fairly good	59	53–64	
	Fairly poor	57	49–65.5	
	Poor	58.5	51–62.5	
Preferred mode of course delivery after the pandemic	Online	53	47.5–60	<0.001
	On-campus	60	55–65.75	
	Blended	60	53.5–66	
Preferred mode of exam delivery	Online	56.5	47–62	0.042
	On-campus	58.5	53–64	
	Both are equal	60	52–66	
Preferred mode of lab delivery	Online	51.5	44.25–60	0.011
	On-campus	59	53–64	
	Both are equal	58	46.75–66	
Preferred mode of lecture delivery	Online	56	49–63	0.1
	On-campus	59	52–64	
	Both are equal	59	54–66	
Preferred mode of asking questions during classes	Online	57	50.25–62	0.005
	On-campus	58	51–64	
	Both are equal	61	58–66.25	

Motivation levels are given as the full-scale result of the updated Strength of Motivation for Medical School (SMMS-R) questionnaire, with a total score from 15–75. Continuous variables are given as median and interquartile range (IQR).

**Figure 1:**
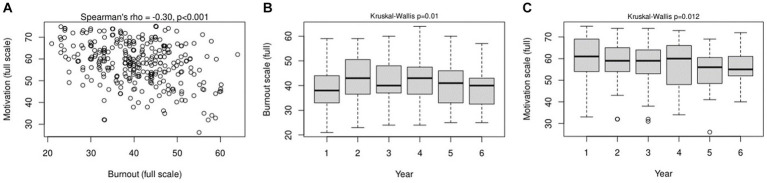
Correlation between student burnout and motivation levels **(A)**, in addition to burnout **(B)**, and motivation **(C)** levels among the six years of studies in our medical school.

Interestingly, burnout levels were inversely correlated with the motivation to study in our medical students at a significant level. This was found when the full OLBI-S and SMMS-R scales were compared (rho = −0.30, *p* < 0.001; Figure 1A), in addition to their subscales (Supplementary Figure S1). Furthermore, when students from different years of study were compared, the burnout levels differed (*p* = 0.01), being the highest in year two (median: 43 [36.75–50.25]) and year four (median: 43 [36.50–47.50]) (Figure 1B). Similarly, motivation levels differed as well (*p* = 0.012), being the lowest in years five (median: 56 [48.5–60.5]) and six (median: 55 [52.0–61.0]) (Figure 1C).

In multivariable analysis, being a female (*b* = 2.22, *p* = 0.016), studying in the fourth year (*b* = 2.54, *p* = 0.049), a perceived beginner/intermediate technology level (vs. advanced/expert; *b* = 2.05, *p* = 0.032), and a perceived poor school support system during the pandemic (*b* = 6.35, *p* < 0.001) were independently associated with higher burnout levels in our medical students (Table 2). Regarding motivation, this was shown to be reduced in the second to sixth year of study compared to the first, yet the result was significant only in the fifth-year vs. the first (*b* = −5.17, *p* = 0.019). Similar to the burnout, a perceived poor school support system during the pandemic was also independently associated with a reduced motivation to study medicine (*b* = −3.09, *p* = 0.01; Table 3). Item missingness was low in both models (24/333, 7.2%), we therefore limited ourselves to a complete case analysis.

**Table 2:** Multivariable linear regression model of factors predicting higher burnout levels in medical students during the COVID-19 pandemic.

Variable	Beta	SE	*t*	*p*-value
Females	2.22	0.92	2.41	**0.016**
2nd Year (vs. 1st)	0.09	1.73	0.05	0.96
3rd Year (vs. 1st)	1.96	1.31	1.49	0.14
4th Year (vs. 1st)	2.54	1.28	1.98	**0.049**
5th Year (vs. 1st)	1.64	1.79	0.91	0.36
6th Year (vs. 1st)	−0.87	1.66	−0.52	0.6
German, Greek, or other as first language (vs. English)	−1.67	1.58	−1.06	0.29
Beginner/intermediate technology level (vs. advanced/expert)	2.05	0.95	2.15	**0.032**
Financial condition worsened	1.86	0.96	1.95	0.05
Health issues reported	1.85	1.00	1.86	0.06
Extracurricular responsibilities	0.99	0.93	1.07	0.29
Poor school support system (vs. good)	6.35	0.99	6.41	**<0.001**

*Adjusted *R*-square = 0.23.

Bold means statistically significant values (*p* < 0.05).

**Table 3:** Multivariable linear regression model of factors predicting motivation to study medicine during the COVID-19 pandemic.

Variable	Beta	SE	*t*	*p*-value
Females	1.13	1.12	1.01	0.31
2nd Year (vs. 1st)	−0.70	2.11	−0.33	0.74
3rd Year (vs. 1st)	−2.39	1.60	−1.50	0.14
4th Year (vs. 1st)	−2.16	1.57	−1.38	0.17
5th Year (vs. 1st)	−5.17	2.19	−2.36	**0.019**
6th Year (vs. 1st)	−3.19	2.03	−1.57	0.12
German, Greek, or other as first language (vs. English)	−3.63	1.90	−1.91	0.06
Beginner/intermediate technology level (vs. advanced/expert)	0.73	1.18	0.62	0.54
Financial condition worsened	−0.50	1.16	−0.43	0.67
Health issues reported	1.02	1.20	0.85	0.4
Extracurricular responsibilities	−2.06	1.13	−1.83	0.07
Poor school support system (vs. good)	−3.09	1.19	−2.59	**0.01**

*Adjusted *R*-square = 0.04.

Bold means statistically significant values (*p* < 0.05).

## Discussion

In this cross-sectional study, we first showed that higher burnout levels were significantly correlated with a lower motivation to study medicine. Furthermore, burnout levels were the highest in the 2nd and 4th year students, with a significant difference in the 4th vs. the 1st year of studies. Female sex, the 4th year of studies, a less advanced technology level, and a suboptimal school support system were independent predictors of higher burnout levels among medical students during the pandemic. Concurrently, motivation was the lowest in the last 2 years of studies, while studying in the 5th year vs. the 1st and a suboptimal school support system were significant predictors of a reduced motivation to study medicine.

Burnout has been reported to be a significant issue affecting medical students and practitioners worldwide (23, 24). Similar to what is published in the literature in relation to lifestyle (25–30), our study also revealed a significant association of higher burnout levels with unhealthy behavior such as lack of physical exercise, smoking, poor eating habits, weight gain, poor sleep quality, and intensive social media use. A meta-analysis performed recently, pooling relevant studies until November 2021, showed that burnout presented in around one out of three medical students [pooled prevalence: 37.23% (32.66, 42.05%)] (24); notably, females and more senior students were more likely to experience it. Burnout prevalence was higher in some countries, for example USA [49.99% (45.12, 54.86%)], than others, for example Canada [27.56% (18.83, 38.42%)] (24). Meanwhile, another survey delivered to USA-based medical students, residents/fellows, and early career specialists revealed that all three groups were statistically more likely to experience burnout than the population control group enrolled in the study (*p* < 0.0001) (31). In a study conducted in a Chinese University before the pandemic, Liu et al. showed that the more advanced medical students exhibited higher burnout risk and lower engagement, compared to the ones studying in the first 2 years (32). Similarly, Hansell et al. reported that burnout became significantly more common in medical students at the end, compared to the beginning of their studies; of interest, emotional exhaustion spiked after years one and three, in their 4 years medical program (33). In our study, we found that burnout levels were higher in the 2nd and the 4th year students of our 6 year program. The 2nd year curriculum introduces students to clinical medicine through laboratory sessions with extensive task training and patient simulation. As such, the COVID-19 pandemic required shifting the delivery of this content from hands-on sessions to online learning modules. Thus, we believe that this transformation was suboptimal for our students, causing additional stress. Similarly, the 4th year students’ curriculum introduces them to clinical training, which was also affected from the pandemic. We assume that the lack of prior significant clinical exposure at this point also acted as an important stressor, leading to increased burnout rates. Consequently, our findings support that the study years with the most significant changes in the curriculum delivery method within our school were the ones mostly affected by the COVID-19 pandemic, in terms of burnout levels. Of interest, another relevant study performed in Cyprus during this period from Zis et al. compared the survey data with the ones collected in the same University before the pandemic and reported similar burnout levels (around 18%) between the two periods; of interest, burnout levels statistically increased in the 6th year and decreased in the 4th year medical students in the post- vs. the pre-COVID-19 era (20).

As shown in this study, female medical students were more prone to experiencing higher burnout levels than males, which is consistent with the literature (24, 34). Similarly, we have shown in our previous study that being a female was also linked with enhanced perceived stress levels while studying medicine during the COVID-19 pandemic (8). Kheirallah et al. performed a cross-sectional study involving medical students in Jordan at the onset of the pandemic and reported enhanced levels of negative and reduced levels of positive emotions among them. Of interest, self-reported worry, depression, and panic, in addition to dropped levels of joy and excitement, were found significantly more often in females rather than males (35). Likewise, in another study from the same country, obsession towards pandemic measures was also more prevalent in female compared to male medical students at a significant level (36).

Regarding motivation to study medicine during the pandemic, only a few published studies have so far been published. Bolatov et al. reported that motivation of their first year medical students was enhanced as soon as blended learning (mixture of on-campus and online sessions) took place instead of pure online learning, pointing to the importance of academic life satisfaction and university belongingness (37). In a qualitative study performed in Japan, medical science students were generally motivated to study during the pandemic, especially at its beginning, due to various scientific and humanitarian reasons. However, the prolonged pandemic demotivated a few of them, while this drop in their motivation was associated with the online learning and established lockdown policies (38). In our study, the decreased motivation levels shown in the last 2 years may at least partially be explained by the disruption of clinical training, in addition to the reduction of physical exposure to the University environment which applied to all 6 years. In contrast to our medical students at the beginning of their studies, 5th and 6th year students might have felt more uncertain and not sufficiently prepared for their professional future in a rapidly changing world triggered by the COVID-19 pandemic. We also find the relationship between the mode of delivery and student motivation in our study very interesting. Consistently, students who preferred online only activities had a lower motivation when compared to students preferring on campus didactic activities or were indifferent about the delivery mode. However, this does not mean that the mode of delivery affects student motivation; students with a lower motivation might find online activities less demanding and more comfortable, whereas highly motivated students often prefer the traditional on-campus curriculum or are willing to adapt to the requirements imposed by special circumstances in order to achieve their goal. For this reason, we chose not to include these variables in our multivariable linear regression models.

This study has some important limitations. First, we used a cross-sectional design, which did not allow the investigation of a potential causal association among burnout and motivation. Additionally, our findings are based on a survey delivered to students of a single medical school, while the fact this was an anonymous survey could have implemented bias in the students’ answers. Unfortunately, we lacked relevant pre-pandemic data, thus it was not possible to compare them with our current findings, thus assess more effectively the pandemic effect on burnout and motivation to study medicine.

In conclusion, the COVID-19 pandemic created multiple challenges for both medical students and educators, impacting their perceived stress, burnout, and motivation levels. In our medical student population, higher burnout levels were correlated with lower motivation to study medicine, while the latter was the lowest in the last 2 years of medical studies. Both burnout and motivation levels differed significantly among the 6 years of our school. Further research would be needed to define the impact of the COVID-19 pandemic on the burnout and motivation of medical students and develop strategies to minimize any negative impact of this period in the years to come; this would further add to the preparedness for future health crises.

## Data availability statement

The original contributions presented in the study are included in the article/Supplementary material, further inquiries can be directed to the corresponding authors.

## Ethics statement

The studies involving human participants were reviewed and approved by Cyprus National Bioethics Committee (2021.01.79). The patients/participants provided their written informed consent to participate in this study.

## Author contributions

IN, ST, DM, EJ, CP, and CT contributed to the study conception and design. IN and ST were responsible for data collection. TL, DN, and IN performed the data analysis and prepared figures and tables. CM, LP, and IN wrote the original draft of the manuscript. CM, LP, TL, DM, ST, CP, CT, EJ, DN, and IN contributed to reviewing and editing of the manuscript. IN and DN were responsible for supervision. All authors contributed to the article and approved the submitted version.

## Conflict of interest

DM and IN were employed by Kenhub GmbH, Leipzig, Germany.

The remaining authors declare that the research was conducted in the absence of any commercial or financial relationships that could be construed as a potential conflict of interest.

## Publisher’s note

All claims expressed in this article are solely those of the authors and do not necessarily represent those of their affiliated organizations, or those of the publisher, the editors and the reviewers. Any product that may be evaluated in this article, or claim that may be made by its manufacturer, is not guaranteed or endorsed by the publisher.
