# Supplementary material for: Burnout and motivation to study medicine among students during the COVID-19 pandemic
Source: Front Med (Lausanne). 2023 Aug 28;10:1214320. doi: 10.3389/fmed.2023.1214320 (PMC10493291; doi:10.3389/fmed.2023.1214320)

**Table S1**. Univariate analysis of the effect of student and pandemic related factors on medical student burnout during the COVID-19 pandemic. Burnout levels are given as the full-scale result of the Oldenburg Burnout Inventory questionnaire adapted for academic environments (OLBI-S) questionnaire, with a total score from 16-64. Continuous variables are given as median and interquartile range (IQR).

|  | **Burnout OLBI-S (Full Scale)** | | | |
| --- | --- | --- | --- | --- |
| **Variable** | **Categories** | **Median** | **IQR** | ***P*-value** |
| Physical exercise frequency | Daily  A few times weekly  A few times monthly  Rarely/never | 37  39  42  42 | 26-44  33-44  34-48  38-47 | **<0.001** |
| Alcohol drinking frequency | Daily  A few times weekly  A few times monthly  Rarely/never | 42  40  39  40 | 34.5-50.25  35-46  33-45.5  34-47 | 0.52 |
| Smoking | ≥1 pack/day  1 pack/2-4 days  1 pack/week  Rarely/never | 42  44  40  40 | 39-51  39.75-49  34-46  33-46 | **0.03** |
| Body weight | Gained weight  Lost weight  Maintained weight | 43  40  39 | 38-49  35-44  32-46 | **0.002** |

| Sleep quality | Very good  Fairly good  Fairly poor  Poor | 37  39  42  44 | 30.5-43  33-45  37-48  38.75-47 | **0.004** |
| --- | --- | --- | --- | --- |
| Financial situation | Improved  Was stable  Deteriorated | 34  39  43 | 26.5-41  33-45  38-48 | **<0.001** |
| Health issues / negative personal life events | Yes  No  Prefer not to say | 43  39  42 | 37-50  32.75-44.25  38-46.5 | **<0.001** |
| Extracurricular responsibilities | Yes  No | 42  39 | 35.25-47  33-46 | **0.013** |
| Social media use | >3hours/day  1-3 hours/day  <1hour/day | 43  39  39 | 37-49  33-44  31-45 | **0.002** |
| Time spent in class preparation | >5 hours/day  3-5 hours/day  1-3 hours/day  <1 hour/day | 41  38  40  44 | 33-47  31-44  35-45  37-51 | **0.009** |
| Perceived support from faculty/advisors | Very good  Fairly good  Fairly poor  Poor | 37  38  44  47.5 | 30-41.5  33-43.75  39-47  43.75-54.25 | **<0.001** |
| Mother language | Greek  English  German  Other | 39  44  44  45 | 33-44  37.25-48.75  36-47  35.75-50 | **0.009** |
| Self-reported familiarity with technology | Expert  Advanced  Intermediate  Beginner | 37  40  43  35 | 30-44  33-46  38-47  31.5-35 | **0.002** |

**Figure S1**. Correlations between the different subscales of the burnout and motivation questionnaires used in this study.


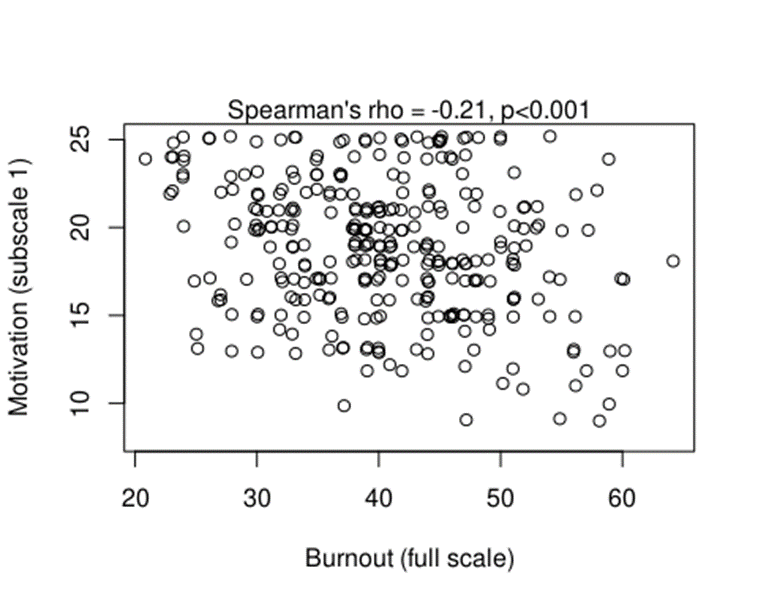


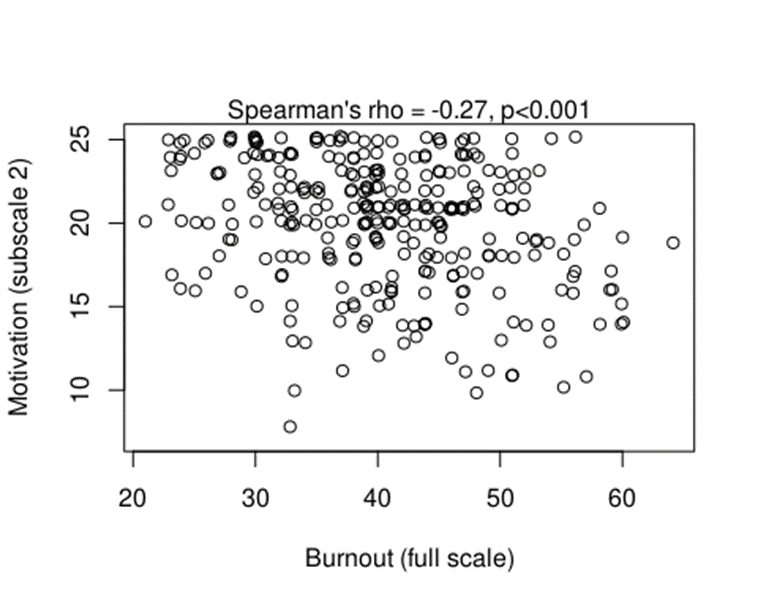


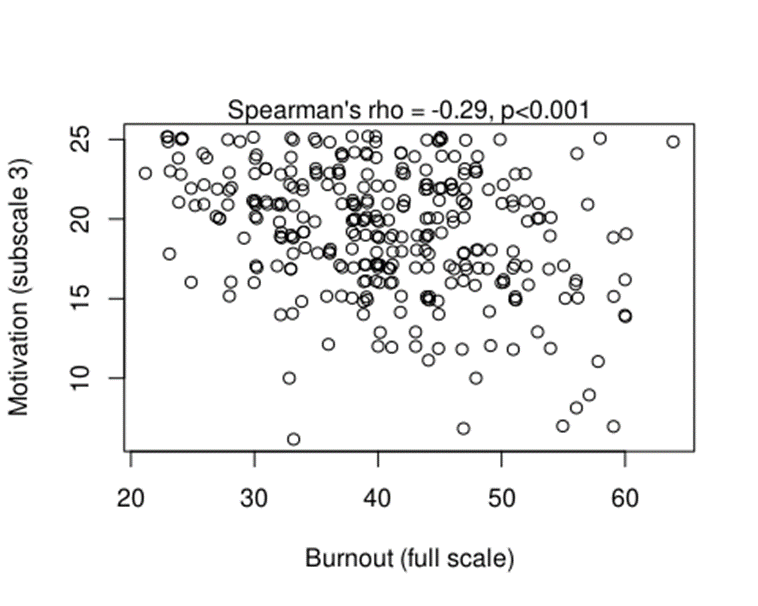


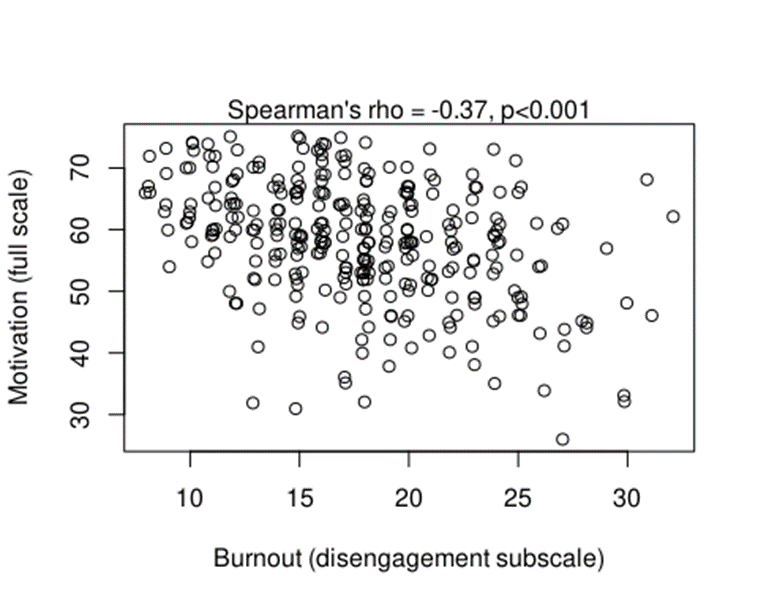


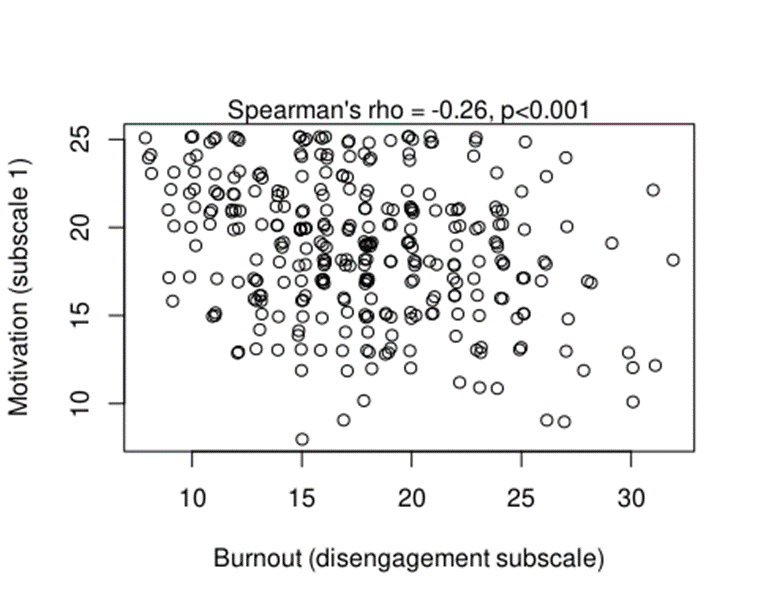


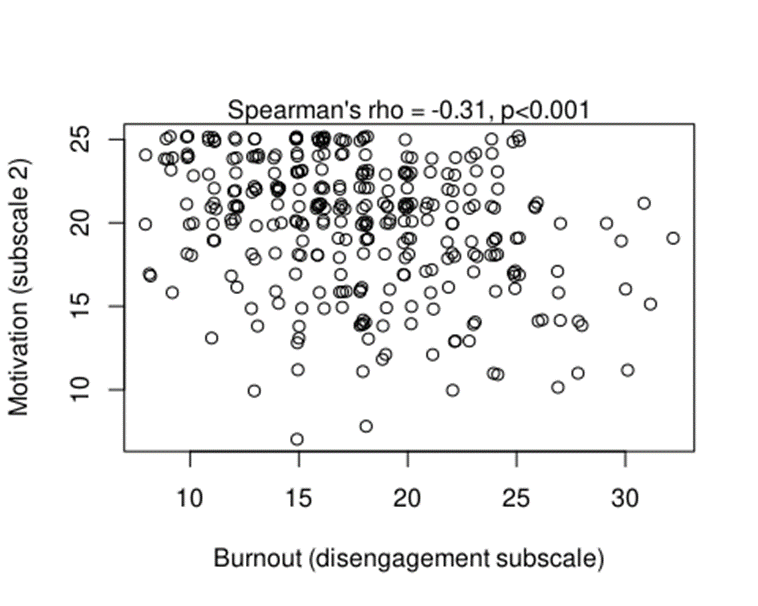


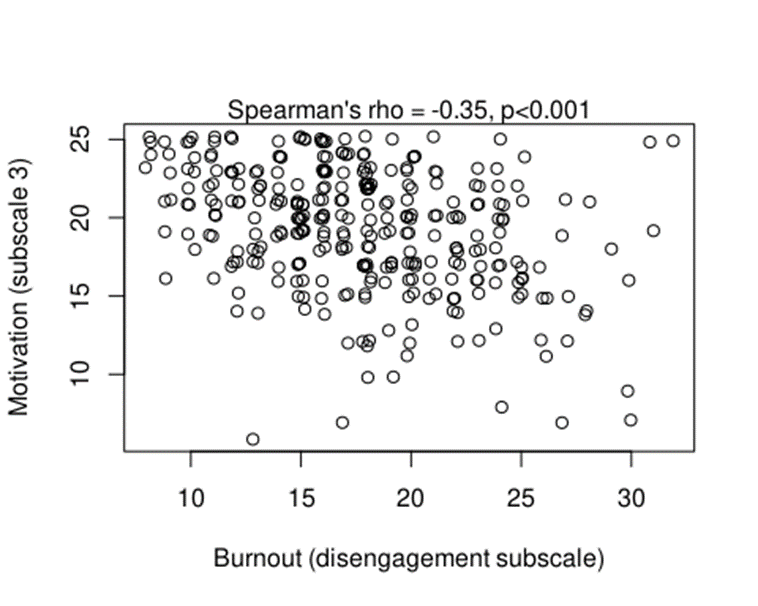


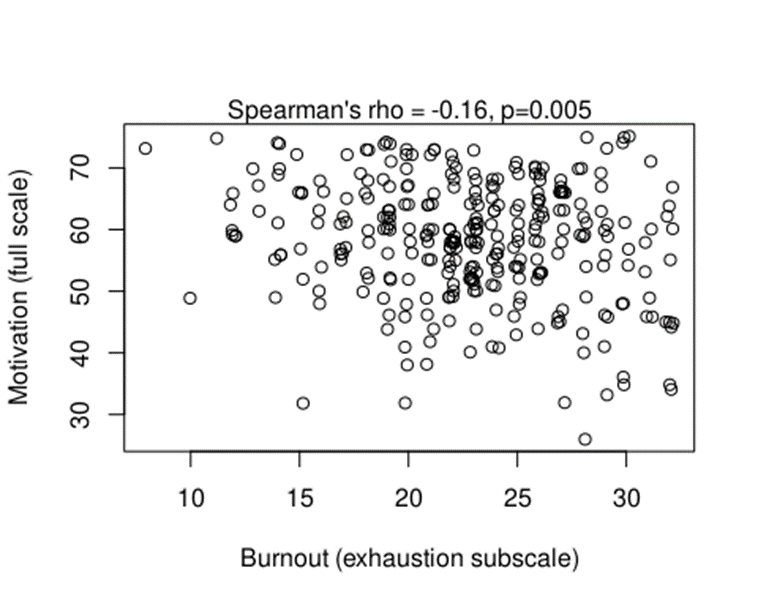


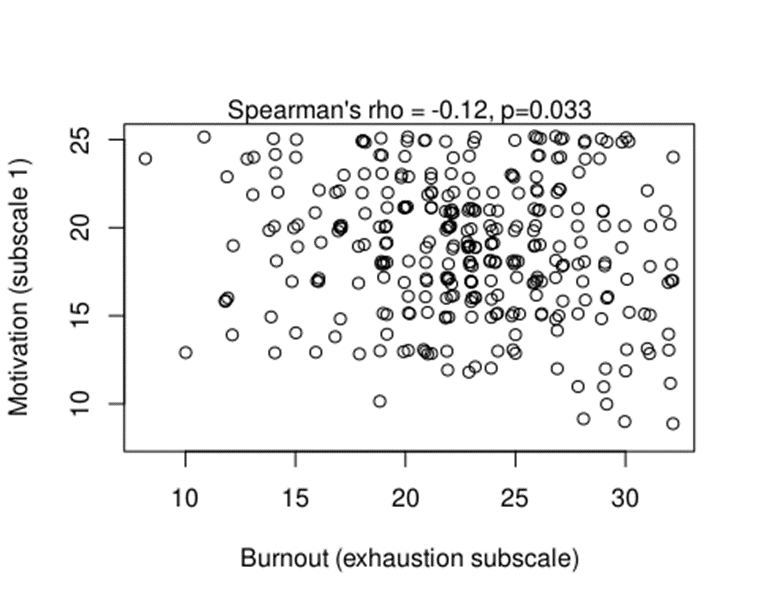


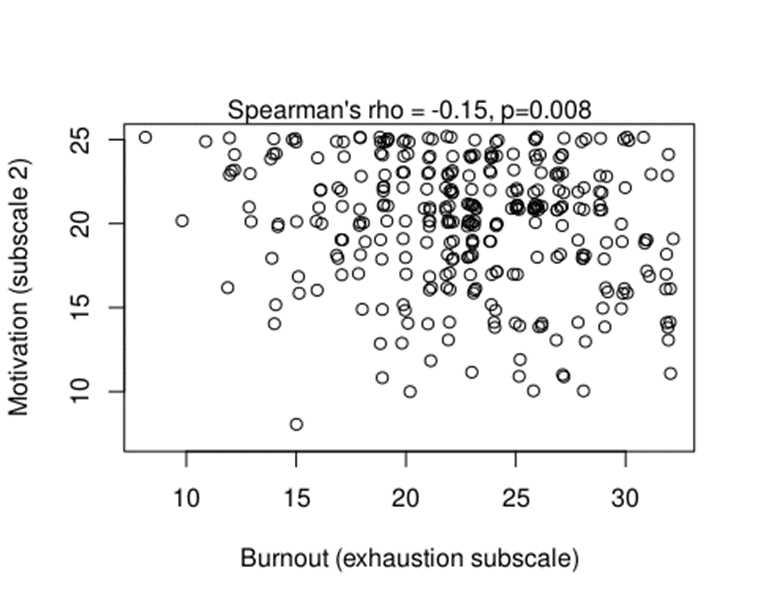


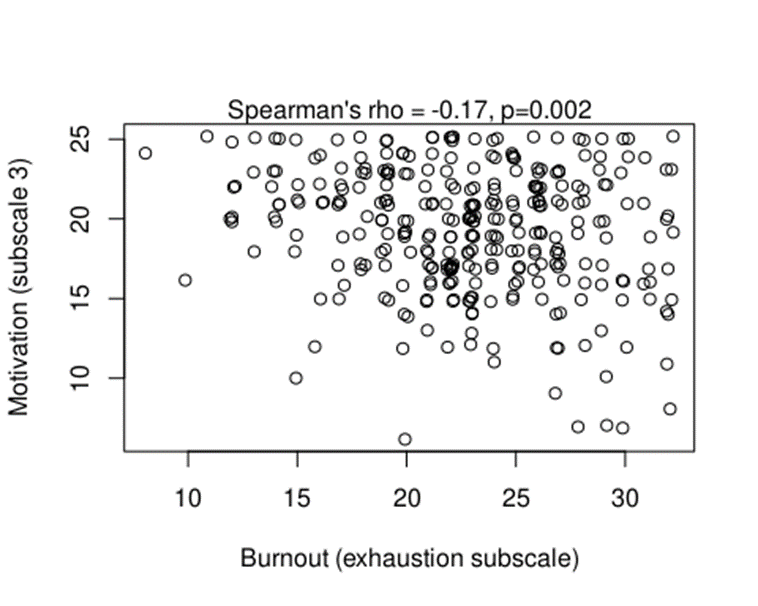

Supplement: Supplementary file 1 [file Data_Sheet_1.docx]
